# A Systematic Review of the Molecular Mechanisms Involved in the Association Between PCOS and Endometrial and Ovarian Cancers

**DOI:** 10.1111/jcmm.70312

**Published:** 2024-12-25

**Authors:** M. Zanjirband, M. H. Nasr‐Esfahani, N. J. Curtin, Y. Drew, S. Sharma Saha, P. Adibi, J. Lunec

**Affiliations:** ^1^ Department of Animal Biotechnology, Reproductive Biomedicine Research Center Royan Institute for Biotechnology, ACECR Isfahan Iran; ^2^ Translational and Clinical Research Institute, Newcastle University Cancer Centre, Faculty of Medical Sciences Newcastle University Newcastle Upon Tyne UK; ^3^ BC Cancer Vancouver and University of British Columbia Vancouver British Columbia Canada; ^4^ Department of Pathology and Experimental Cancer Research Semmelweis University Budapest Hungary; ^5^ Biosciences Institute, Newcastle University Cancer Centre, Faculty of Medical Sciences Newcastle University Newcastle Upon Tyne UK

**Keywords:** biomarker, EC, molecular signature, OVCA, PCOS

## Abstract

Polycystic ovary syndrome (PCOS), a major cause of female infertility, affects 4%–20% of reproductive‐age women. Metabolic and hormonal alterations are key features of PCOS, potentially raising the risk of endometrial (EC) and ovarian (OVCA) cancers. This systematic review aims to summarise the proposed molecular mechanisms involved in the association between PCOS and EC or OVCA. This is achieved by conducting a thorough literature review and utilising specific search terms to identify all relevant studies published in English from 2010 to December 2022. PRISMA was followed, and the protocol was registered on PROSPERO (CRD42022375461). The QUADAS‐2 tool and Review Manager Software were employed to evaluate study quality and risk of bias respectively. Forty‐five eligible studies were selected with molecular signatures based on genomic, transcriptomic, metabolomic, proteomic and epigenetic analyses. Genes and their products deregulated in EC and/or OVCA were identified, including *BRCA1*, *MLH1*, *NQO1* and *ESR1*, which were also deregulated in PCOS. Serum levels of IGF1, IGFBP1, SREBP1 and visfatin in women with PCOS were also identified as potential biomarkers of enhanced EC risk. Salusin‐β serum levels in individuals with PCOS were identified as a potential biomarker for increased risk of OVCA. Gene signature–based drug repositioning identified several drug candidates: metformin, fenofibrate, fatostatin, melatonin, resveratrol and quercetin, some already established and prescribed for PCOS. In conclusion, this study provides a strong basis for further research to confirm the identified molecular signatures and associated causal links for potential therapeutic prevention strategies for EC and OVCA in women with PCOS.

## 1 Introduction

Polycystic ovarian syndrome (PCOS) is the most common endocrine‐gynaecology disorder with variable prevalence, 4%–20%. Diagnosis requires the presence of two of the following criteria: hyperandrogenism, ovulation dysfunction and polycystic ovarian morphology, as per the Rotterdam PCOS diagnostic criteria [1]. PCOS is a complex condition with multiple potential contributors, including genetic and epigenetic mechanisms, insulin resistance, hormonal imbalances, inflammation, oxidative stress and obesity, which gradually may lead to cancer over time [2, 3].

EC (endometrial cancer) and OVCA (ovarian cancer) are two common gynaecological malignancies in women with poor prevention strategies and survival rates [4, 5, 6]. The association of PCOS with EC and OVCA was first reported in 1949 [4] and 1996 [5] respectively. The risk of EC in individuals with PCOS has been reported to be 20%–37% compared to the global 11.28% incidence rate of EC in non‐PCOS women. Regarding OVCA, 37% of women with PCOS are at risk of developing OVCA compared to 3.0%–11.4% in non‐PCOS women worldwide [4, 7, 8]. Subjects with PCOS are at 2.7‐ to 4‐fold higher risk of EC relative to non‐PCOS women [8, 9, 10, 11, 12, 13, 14]. Several studies have reported key findings as possible candidates for contribution to the link between PCOS and EC or OVCA [12, 15, 16, 17, 18].

An Australian case–control study was performed to evaluate the correlation of PCOS with EC risk in women aged less than 50 years. The results demonstrated a significantly positive association between PCOS and EC (OR = 4.0, 95% CI = 1.7–9.3). The trend was similar when data were adjusted for BMI, although it was weakened (OR = 2.2, 95% CI = 0.9–5.7) [19]. A recently published study investigated the correlation of PCOS with various types of cancer in a large population‐based cohort of 3,493,604 Swedish females aged 15–50 years. A correlation was observed between PCOS and overall cancer risk, in particular, excess cancer risks were detected at specific sites following fully adjusted models, including endometrium (HR, 2.62; 95% CI, 1.58–4.35) and ovary (HR, 2.16; 95% CI, 1.30–3.59) [20]. A Danish study of 12,070 women with PCOS found that the risk of EC is higher in individuals with PCOS (*N* = 16, SIR = 3.9; 95% CI = 2.2–6.3). However, the overall incidences of OVCA (*N* = 10, SIR = 1.8; 95% CI = 0.8–3.2) and breast cancer (*N* = 59, SIR = 1.1; 95% CI = 0.8–1.4) were the same as for women in the general population [14]. The link between PCOS and EC was also reported in another study of 117 Korean women with PCOS. Endometrial hyperplasia and EC rates in this cohort were 21.4% and 1.7% respectively [21].

PCOS and EC or OVCA may be linked due to common genetic and epigenetic changes, suggesting a possible common causal link between these diseases. The known predisposing factors like obesity, hyperinsulinemia, chronic anovulation, infertility and diabetes increase the risk of both PCOS and EC [7, 8, 9]. It is thought that chronic and unopposed oestrogen exposure to the endometrium is the main mechanism involved in the elevated risk of EC [3, 4, 9, 10] and OVCA [6] in individuals with PCOS.

Despite the epidemiological evidence reporting an association between PCOS and EC or OVCA, insufficient mechanistic evidence regarding such a correlation has been reported. This systematic review aims to identify these mechanisms and potential biomarkers for the prevention, early detection and treatment management of these cancers.

## 2 Methodology

The present study was conducted according to the Preferred Reporting Items for Systematic Review and Meta‐Analysis (PRISMA) guidelines [22]. The protocol for this systematic review was registered on PROSPERO, an international database of prospectively registered systematic reviews in health and social care. The registration number for this study is CRD42022375461, and the full protocol is available in full on the NIHR HTA (National Institute for Health and Care Research, Health Technology Assessment) programme website (https://www.crd.york.ac.uk/prospero/display_record.php?ID=CRD42022375461).

### 2.1 Literature Search and Data Abstraction

The PubMed and POPLINE databases were searched between 2010 and December 2022 using specific terms defined in Table S1. The search was limited to articles in the English language and full text. The inclusion criteria were research articles and studies scored as moderate or high quality (with a score of 6 or higher) based on the Study Quality Assessment Tools (https://www.nhlbi.nih.gov/health‐topics/study‐quality‐assessment‐tools). The study focused on identifying genetic and epigenetic similarities between individuals with PCOS and those with endometrial or ovarian cancer. Only high‐quality research articles using transcriptome sequencing and proteomic and epigenomic technologies were included in the study. Animal studies, reviews, abstracts, poor‐quality studies and duplicated data were excluded. Studies that did not compare against healthy controls or individuals with PCOS were also excluded.

The study titles and abstracts underwent a review and screening by two coauthors (MZ and PA), who were blinded to each other's decisions. Disagreements were resolved through group meetings and discussions. Microsoft Excel 2010 was used to record selected studies and their scores according to the Study Quality Assessment Tools. Duplicated articles were identified and removed. The original pdf files were located via direct online links. References were manually searched for potentially relevant studies. The search findings were double‐checked by one coauthor (PA).

### 2.2 Data Extraction and Quality Assessment

Using a standard form, two investigators (MZ and PA) independently extracted data from eligible studies. This included population characteristics, method and inclusion/exclusion criteria. A third author (JL) reviewed eligibility criteria in case of disagreement [22].

The QUADAS‐2 (Quality Assessment Tool for Diagnostic Accuracy Studies) tool was employed by two independent authors (MZ and PA) to evaluate the quality of studies and determine bias risk [23]. Each study was scored from 0 to 14, with good studies scoring 10–14, fair studies 6–9 and poor studies 0–5. One coauthor (PA) screened studies, and discrepancies were discussed with a third reviewer (JL). The assessment used Review Manager Software, Version 5.4 (The Cochrane Collaboration, London, UK, 2020).

### 2.3 Strategy for Data Synthesis

Data synthesis involved tabular and narrative presentations of analysis findings. Narrative synthesis was chosen due to varied studies, and the PRISMA statement was followed [22]. Preliminary synthesis involved a thematic analysis presented in tabular form. Results were structured into themes and discussed. Finally, a narrative synthesis was performed within a framework.

## 3 Results and Discussion

**FIGURE 1:**
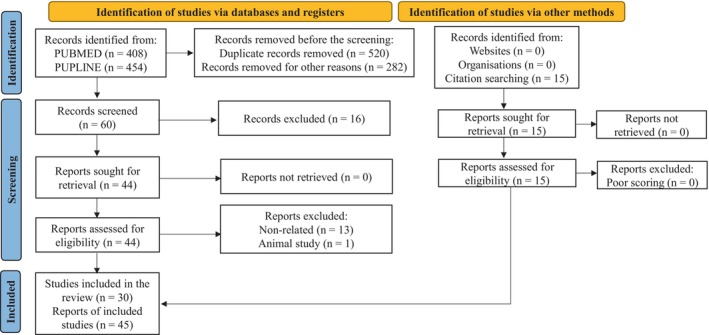
A flowchart summarising the selection process of the primary studies.

The literature search of PUBMED and POPLINE resulted in 878 citations. After excluding irrelevant articles (833), the remaining articles (45) were analysed. Out of these, 10 were extracted from PUBMED, 7 from POPLINE, 13 from both databases and 15 from manual search. The descriptive information of the eligible studies has been presented in Table S2. The results have been categorised into genomics‐ (Table 1, 6 studies), transcriptomics‐ (Table 2, 9 studies), proteomics‐ and metabolomics‐ (Table 3, 12 studies) and epigenetics‐based (Table 4, 7 studies) markers. A flow diagram of the systematic review is shown in Figure 1.

**TABLE 1:** Summary of genomics‐based analyses.

Author	Case No	Control No	Technology	Common genes	*p‐value*	*p*‐adjusted^a^
Sekar et al. [2]	PCOS (10)	Control (4)	Cytogenic	Chromosome aberrations	*< 0.001*	—
Jiao et al. [5]	PCOS‐IRM (10)	PCOS‐RM (10)	WES	Multiple point mutations in *BRCA1*, *MLH1*	NA	NA
Wang et al. [24]	EC (96)	Control (192)	PCR Sequencing	rs2479106 (G/A) rs13405728 (G/A)	*0.076* *0.384*	*0.008* *0.036*
Day et al. [25]	PCOS (2045)	Control (98,886)	GWAS	*HER4, YAP1, THADA, FSHB, RAD50, KRR1*	*< 5* * ×* *10* ^*−8*^	—
Xiong et al. [26]	^b^	^c^	PCR‐RFLP	MTHFR C677T & MTHFR A1298C polymorphisms	*< 0.0001*	—
Bhanoori et al. [27]	PCOS (110)	Control (130)	PCR Sequencing	*TP53* (rs1042522 G/C) & *BRCA1* (rs71361504 −/GTT, rs3092986 T/C) polymorphisms	*< 0.05* or *< 0.01*	—

Abbreviations: *BRCA1*, breast cancer type 1 susceptibility protein; EC, endometrial cancer; *FSHB*, follicle‐stimulating hormone subunit beta protein; GWAS, genome‐wide association study; *HER4*, human epidermal growth factor receptor 4; *MLH1*, MutL homologue 1; MTHFR, methylenetetrahydrofolate reductase; NA, not applicable; No, number; PCOS‐IRM, polycystic ovarian syndrome‐irregular menstruation; PCOS‐RM, polycystic ovarian syndrome‐regular menstruation; PCR, polymerase chain reaction; PCR‐RFLP, polymerase chain reaction‐restriction fragment length polymorphism; *RAD50*, restriction site‐associated DNA50; *THADA*, thyroid adenoma associated; WES, whole exome sequencing; *YAP1*, yes‐associated protein‐1.

^a^ The *p‐*value was adjusted by BMI (body mass index) and age.

^b^ MTHFR C677T, PCOS (2337); MTHFR A1298C, PCOS (784).

^c^ MTHFR C677T, control (2469); MTHFR A1298C, control (854).

**TABLE 2:** Summary of mRNA‐ and transcriptomics‐based analyses.

Author	Case No	Control No	Technology	Common DEGs	*p‐value*
Jiao et al. [5]	OVCA (379)	PCOS‐IRM (10) & PCOS‐RM (10)	Real‐time PCR array	12 upregulated genes^a^ *Downregulated GADD45G*	*< 0.05*
Kori et al. [7]	PCOS (15) Endometriosis (10) OVCA (21)	Non‐PCOS (14) Non‐endometriosis (10) Non‐OVCA (22)	Integrated & comparative (multistage) analysis	6 upregulated genes^b^ 31 downregulated genes^a^	*< 0.01*
Shafiee et al. [8]	PCOS (34) EC (34)	Healthy control (34)	qRT‐PCR ELISA	3 Mutual DEGs^c^	*< 0.01*
Miao et al. [9]	PCOS (6) EC (24)	Healthy control (6) Normal endometrium (3)	Microarray data^d^	10 Shared DEGs^e^	*< 0.05*
Shafiee et al. [13]	PCOS (34) EC (34)	Non‐PCOS women without EC (34)	qRT‐PCR ELISA	Upregulated *SREBP1*	*< 0.0001*
Atiomo et al. [28]	PCOS (26), EC (25)	Non‐PCOS without EC (25)	RNA sequencing qRT‐PCR IHC	RNAseq: 12 Upregulated, 82 downregulated TCGA validation: 14^a^ qRT‐PCR: 3^f^ IHC: Upregulated NQO1	*< 0.05* *< 0.0001*
Desai et al. [29]	Obese PCOS (14)	Obese control (15)	Bioinformatic & microarray analysis	5 DEGs^g^	*< 0.05*
Zhang et al. [30]	OVCA specimen (57)	Matched adjacent normal tissues (57)	qRT‐PCR ELISA	Overexpressed salusin‐β	*< 0.05*
Zou et al. [31]	TCGA (374 OVCA) GSE140082 (380 OVCA) GSE34526 (PCOS 7)	GTEX database (88 normal ovary), GSE34526 (control 3)	RNA sequencing Microarray qRT‐PCR	8 Upregulated genes^h^ 4 Downregulated genes^i^	*< 0.05*

Abbreviations: DEGs, differentially expressed genes; EC, endometrial cancer; EEC, endometrial epithelial cell; ELISA, enzyme‐linked immunosorbent assay; EMSC, endometrial mesenchymal stem cell; ESF, endometrial stromal fibroblasts; PCOS, polycystic ovarian syndrome; qRT‐PCR, quantitative real‐time polymerase chain reaction.

^a^ DEGs > 10 are reported in the Table S3.

^b^ *COX1*, cytochrome c oxidase; *CYCS*, cytochrome c; *ICAM1*, intercellular adhesion molecule 1; *SRGN*, serglycin; *STK17B*, serine/threonine kinase 17B; *TTC39B*, tetratricopeptide repeat protein 39B.

^c^ *IGF1*, insulin‐like growth factor 1; *IGFBP1*, Insulin‐like Growth Factor Binding Protein; *PTEN*, Phosphatase and TENsin homologue.

^d^ Microarray data were retrieved from the GEO (gene expression omnibus) database. The GSE48301 dataset was analysed using the Agilent GPL6244 platform [HuGene‐1_0‐st], and the GSE115810 dataset was analysed using the Agilent GPL96 platform [HG‐U133A].

^e^ *ESR1*, oestrogen receptor 1; *GTF2H3*, general transcription factor IIH subunit 3; *JUN*, Jun proto‐oncogene AP‐1 transcription factor subunit; *MRPL16*, mitochondrial ribosomal protein L16; *MRPL17*, mitochondrial ribosomal protein L17; *MRPL22*, mitochondrial ribosomal protein L22; *MRPS11*, mitochondrial ribosomal protein S11; *RPL26L1*, ribosomal protein L26 Like 1; *RPL37A*, ribosomal protein L37a; *UBE2I*, ubiquitin conjugating enzyme E2 I.

^f^ *NQO1*, NAD(P)H quinone oxidoreductase 1; *TP53*, tumour suppressor p53; *GJB2*, gap junction beta 2.

^g^ *CXCL2*, chemokine (C‐X‐C Motif) Ligand 2; *FGF10*, fibroblast growth factor 10; *HGF*, hepatocyte growth factor; *HHIP*, hedgehog‐interacting protein; *WNT2*, wingless‐type MMTV integration site family, member 2.

^h^ CXCL11, C‐X‐C motif chemokine ligand 11; GALNT6, polypeptide N‐acetylgalactosaminyltransferase 6; IL27RA, interleukin 27 receptor subunit alpha; JCHAIN, joining chain of multimeric IgA and IgM; LPAR3, lysophosphatidic acid receptor 3; OR7E14P, olfactory receptor family 7 subfamily E member 14 pseudogene; RNF144B, ring finger protein 144B; STAT1, signal transducer and activator of transcription 1.

^i^ CRISPLD2, cysteine‐rich secretory protein LCCL domain containing 2; NR4A1, nuclear receptor subfamily 4 group A member 1; OGN, Osteoglycin; PTPRD, protein tyrosine phosphatase receptor type D.

**TABLE 3:** Summary of protein‐, proteomics‐ and metabolomics‐based analyses.

Author	Case No	Control No	Technology	Protein/metabolite reporter	*p‐value*
Kori et al. (2016) [7]	PCOS (15) OVCA (21)	Non‐PCOS (14) Non‐OVCA (22)	Integrated & comparative (multistage) analysis	Downregulated^a^ (2) Upregulated^b^ (3) Downregulated^c^ (1) Upregulated^d^ (1)	*< 0.05*
Tian et al. [11]	PCOS (80) PCOS‐EL^f^ (20)	Non‐PCOS (80) PCOS‐NE (40)	ELISA WB	Upregulated^e^ (3) Upregulated^g^ (3)	*< 0.05*
Paulson et al. [32]	Obese‐PCOS (20) Anovulatory obese‐PCOS (11)	BMI‐matched controls (10) BMI‐matched controls (10)	IHC	Upregulated^h^ (1) Upregulated^i^ (2)	*< 0.05* *< 0.05*
Giordano et al. [33]	PCOS (11)	Non‐PCOS (8)	IHC	Downregulated^j^ (5) Upregulated^k^ (1)	*< 0.001* *< 0.001*
Hu et al. [34]	PCOS (22)	Non‐PCOS (17)	IHC, WB	Upregulated AR, TLR2/4, IRF‐7	*< 0.05*
Englert‐Golon et al. (2021) [35]	OVCA (25)	Control (27: 12 healthy control & 15 women with non‐cancerous benign changes)	IHC, WB	The signal was case‐dependent, and no specific pattern was shown.	—
Ramly et al. [36]	8185‐PCOS‐ related proteins	—	MCODE algorithm	PCOS‐related proteins found in OVCA^l^ (5)	—
Zhang et al. [37]	PCOS (10)	Control^m^ (10)	DIGE, MS, IHC, WB	Upregulated^n^ (15) Downregulated^o^ (3)	*< 0.05*
Giordano et al. [1]	PCOS (10)	Non‐PCOS (8)	Electrophoresis	Heparan sulphate	*= 0.03*
Kori et al. [7]	PCOS (15)	Non‐PCOS (14)	Comparative & integrative analysis	Downregulated^p^ (5) Upregulated^q^ (4)	*< 0.05*
Shafiee et al. [10]	PCOS (34) EC (34)	Women with benign gynaecology problems undergoing surgery (34)	LC & HRMS	Monoacylglycerol 24:0 & capric acid	*9.66* *×* *10* ^*−4*^ ^r^ *3.13* *×* *10* ^*−2*^ ^s^
Simoes et al. [38]	PCOS (30)	Eumenorrheic women in the proliferative phase (30)	Fluorometric assay (ELISA‐like)	Hyaluronic acid	*< 0.01*

Abbreviations: AR, androgen receptor; BMI, body mass index; DIGE, differential in‐gel electrophoresis; EC, endometrial cancer; ELISA, enzyme‐linked immunosorbent assay; HRMS, high‐resolution mass spectrometry; IHC, immunohistochemistry; IRF‐7, TLR4‐mediated activation of interferon regulatory factor‐7; LC, liquid chromatography; MS, mass spectroscopy; OVCA, ovarian cancer; PCOS, polycystic ovarian cancer; PCOS‐EL, polycystic ovarian syndrome‐endometrium lesions; PCOS‐NE, polycystic ovarian syndrome‐normal endometrium; TLR2/4, toll‐like receptor 2/4; WB, western blot.

^a^ MAPK1, mitogen‐activated protein kinase 1; *MYC*, Myc proto‐oncogene protein.

^b^ *IKBKE*, inhibitor of nuclear factor kappa B kinase subunit epsilon; *SRC*, SRC proto‐oncogene (nonreceptor tyrosine kinase); *SUMO2*, small ubiquitin‐like modifier 2.

^c^ ESR1, oestrogen receptor 1.

^d^ SUMO1, small ubiquitin‐like modifier 1.

^e^ Visfatin; p‐Ark, phosphorylated serine–threonine protein kinase 1; p‐ERK1/2, phosphorylated extracellular signal‐regulated Kinase 1/2 proteins were upregulated in both serum and endometrium tissue.

^f^ 14 out of 20 PCOS‐EL were PCOS with endometrial hyperplasia, and 6 ones were PCOS with endometrial cancer.

^g^ Visfatin; p‐Ark; p‐ERK1/2 proteins were upregulated in serum and endometrium tissue.

^h^ PRLR, prolactin receptor.

^i^ Ki67, marker of proliferation Ki‐67; PRLR, prolactin receptor.

^j^ Bax, Bcl‐2‐associated X‐protein; Bcl2, B‐cell leukaemia/lymphoma 2 protein; Casp3, caspase 3; Fas, fas cell surface death receptor; FasL, Fas ligand.

^k^ Ki67, marker of proliferation Ki‐67.

^l^ CDKN1B, cyclin‐dependent kinase inhibitor 1B; PPP1CC, serine/threonine‐protein phosphatase PP1‐gamma catalytic subunit; URI1, unconventional prefoldin RPB5 interactor 1; SKP2, S‐phase kinase‐associated Protein 2; BRCA1, breast cancer 1.

^m^ The control group includes women whose one ovary was resected to treat a unilateral benign ovarian teratoma or an ovarian cyst.

^n^ HSP90B1, heat shock protein 90B1; RBP1, retinol‐binding protein 1; PGRMC1, membrane‐associated progesterone receptor component 1; CALM1, calmodulin 1; SET, protein SET; YWHAE, 14–3‐3 protein epsilon; CALR, calreticulin; TUBB, tubulin‐β chain; PTRF, polymerase I and transcript release factor; CIQBP, complement component 1 Q subcomponent‐binding protein; VIM, vimentin; GANAB, neutral alpha‐glucosidase AB; SERPINC1, antithrombin III.

^o^ TPM2, tropomyosin beta chain; ANXA6, Annexin A6; HSPA5, 78 kDa glucose‐regulated protein; LMNA, prelamin‐A/C; ALB, serum albumin.

^p^ Alanine, pyruvate, ganglioside molecules GD1a and GM1b, L‐lysyl‐tRNA(lys).

^q^ Ferricytochrome C, ubiquinol, (5Z,8Z,11Z)‐eicosatrienoyl‐CoA, activation‐PPARα.

^r,s^ Q‐value.

**TABLE 4:** Summary of epigenomics‐based analyses.

Author	Case No	Control No	Technology	Epigenome reporter	*p‐value*
Jiao et al. [5]	PCOS‐IRM (10)	PCOS‐RM (10)	DNA methylation sequencing & microRNA qPCR Assay	Global DNA hypomethylation & differentially expressed miRNAs^a^	*< 0.05*
Che et al. [12]	PCOS (4) Validation: PCOS (30)	(4) (10)	Exosomal miRNA sequencing	Upregulated miR‐27a‐5p	*< 0.05*
Makrinou et al. [39]	PCOS (16)	(16)	DNA methylation microarray	Methylated CpG sites^b^	*< 5.8 × 10^–8^*
McAllister et al. [40]	PCOS (7)	(7)	miRNA global deep sequencing	Downregulated miR‐130b‐3p	*< 0.001*
Zhao et al. [16]	PCOS (20)	(20)^c^	qRT‐PCR	Upregulated miR‐155	*< 0.001*
Hou et al. [18]	PCOS (60)	(60)	qRT‐PCR	Upregulated TMPO‐AS1 Deregulated miR‐355‐5P^d^	*< 0.05* *< 0.05*
Xu et al. [17]	PCOS (21)	(13)	qRT‐PCR	Upregulated circ_FURIN Deregulated miR‐423‐5p	*< 0.05* *< 0.05*

Abbreviations: CpG Sites, regions of DNA where a cytosine nucleotide is followed by a guanine nucleotide in the linear sequence of bases along its 5′ → 3′ direction; TMPO‐AS1, TMPO antisense RNA 1.

^a^ 23 miRNAs were deregulated, amongst which 65.2% are common among PCOS subjects with irregular menstruation and OVCA data, and some of which are involved in oncogenic pathways, such as miR‐205, miR‐520‐e and miR‐22‐3p.

^b^ 106 differentially methylated CpG sites were explored, and they are associated with 88 genes. The core pathway analysis showed ‘molecular mechanism to cancer’ as the most statistically significant pathway with pathologies similar to endometrial cancer.

^c^ Normal ovarian cortical tissues of women with normal menstruation and normal androgen levels.

^d^ Upregulation of premature miR‐355‐5P; downregulation of mature miR‐355‐5p.

### 3.1 Assessment of Quality and Heterogeneity of Studies

**FIGURE 2:**
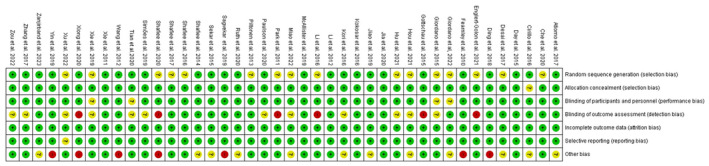
Risk of bias summary: Review of authors’ judgements about each risk of bias item for each included study.

The methodological quality of studies was assessed using the QUADAS‐2 and NIH tools. Data are presented in Figure 2 and Figure S1, showing articles with well‐defined research questions and appropriate methods.

### 3.2 Genomics‐Based Analysis

Recent evidence suggests a correlation between PCOS and EC or OVCA based on genetic background (Table 1).

#### 3.2.1 PCOS and EC

A genome‐wide association study (GWAS) of PCOS in Han Chinese found a strong link between PCOS and three susceptibility loci rs13405728 in *LHCGR* (luteinising hormone/choriogonadotropin receptor), rs13429458 in *THADA* (thyroid adenoma associated) and rs2479106 in *DENND1A* (DENN/MADD domain containing 1A). Then, Wang et al. looked at the genetic structure of women with EC by genotyping these specific PCOS‐related SNPs (single nucleotide polymorphism) using PCR (polymerase chain reaction) and direct sequencing. The results showed that the G allele in rs2479106 in DENND1A and the A allele in rs13405728 in LHCGR were linked with an increased risk of endometrioid adenocarcinoma (*p* < 0.05). *DENND1A* may be involved in EC pathogenesis by regulating neoplasm development in the endometrium. *LHCGR* could contribute to the progression of EC via activation of PKA (protein kinase A), which activates β1 integrin adhesiveness to the basement membrane and induces subsequent secretion of MMP2 (matrix metallopeptidase 2). The minor allele frequencies of rs2479106 and rs13405728 in EC were similar to what has been reported in PCOS (Table 1) [24].

#### 3.2.2 PCOS and OVCA

A large‐scale GWAS study by Day et al. found six significant signals for PCOS at genome‐wide statistical significance (*p* < 5 × 10^−8^). The study included 5184 PCOS cases and 82,759 controls of White European ancestry. Two genes, *ERBB4/HER4* (human epidermal growth factor receptor 4, 2q34) and *RAD50* (restriction site–associated DNA50, 5q31.1), closely linked to cancer, were identified among the six genes. The study found that ERBB4/HER4 had the strongest novel PCOS signal (rs1351592, odds ratio: 1.18 (1.13–1.23), *p* = 1.2 × 10^−12^), indicating a possible role in PCOS pathogenesis. Stimulation of ERBB4/HER4 leads to activation of intracellular RAS and PI3kinase signalling pathways, with various outcomes such as cell proliferation, apoptosis blocking, invasion and metastasis activation. The third signal (rs13164856, *p* = 3.5 × 10^−9^) is located near *RAD50* (5q31.1), producing a protein involved in DNA double‐strand break repair (Table 1) [25].

Several gene polymorphisms that affect metabolic enzymes, such as MTHFR (methylenetetrahydrofolate reductase), might contribute to the development of PCOS and OVCA. However, studies have found inconsistent results, which might be due to small sample sizes, different ethnicities, various matched parameters and genotyping methods. A meta‐analysis of 45 case–control studies focusing on MTHFR gene polymorphisms found that both MTHFR C677T (rs1801133) and MTHFR A1298C (rs1801131) were associated with an increased risk of PCOS, while only MTHFR C677T was linked to a higher risk of OVCA in Asians (TT vs. CT + CC: OR = 2.35, 95% CI = 1.59–3.48; TT + CT vs. CC: OR = 1.49, 95% CI = 1.19–1.86; TT vs. CC: OR = 2.84, 95% CI = 1.88–4.31; T vs. C: OR = 1.49, 95% CI = 1.26–1.77) (Table 1) [26].

One study investigated the relationship of *TP53*, *BRCA1* (breast cancer 1) and *BRCA2* (breast cancer 2) polymorphisms to PCOS incidence in South Indian women [27]. The *TP53* gene (Arg72Pro, rs10425220 G/C) polymorphism indicated a significant increase in the frequency of C/C genotype (*p* = 0.0032) in individuals with PCOS compared to the control group. This trend has also been seen in allele frequency (*p* = 0.0055), which suggests that the ‘C' allele might favour the development of PCOS while the ‘G' allele might provide a minor degree of protection against PCOS. However, the role of the *TP53* codon 72 polymorphisms in cancer has been controversial, and most women with the C/C genotype do not develop OVCA.

Considering *BRCA1* gene (rs71361504−/GTT, rs3092986 T/C) polymorphisms located in the 3′‐UTR regions, the distribution of the genotype (*p* = 0.0058) and allele (*p* = 0.0099) of the rs71361504 (−/GTT) indicated significant differences between the women with PCOS and controls. The frequency of the (−/−) genotype was significantly reduced, whereas the frequency of the (GTT/GTT) genotype was significantly elevated in subjects with PCOS relative to controls (*p* = 0.0058). This polymorphism may significantly impact the transcriptional activation of the *BRCA1* gene. In addition, there were significant differences between the two groups in terms of the frequencies of genotypes (*p* = 0.0103) and alleles (*p* = 0.0014) of the rs3092986 (T/C) polymorphism. However, *BRCA2* gene (rs206118 A/G) polymorphism showed no significant association with PCOS. Noncoding polymorphisms linked to *TP53* and *BRCA1* may be a heritable risk factor for PCOS in South Indian women, suggesting a potential link between PCOS and OVCA (Table 1) [27].

Based on genomics analysis, it is likely that the loci rs13405728 in *LHCGR* and rs1351592 in *ERBB4/HER4* are linked to PCOS and EC or OVCA respectively. Furthermore, noncoding polymorphisms linked to *TP53* and *BRCA1* may be potential links between PCOS and OVCA due to their role in developing both conditions.

### 3.3 mRNA‐ and Transcriptomics‐Based Analysis

Gene expression profile analysis employing microarray and RNASeq data has been extensively used to identify differentially expressed genes (DEGs) (Table 2).

#### 3.3.1 PCOS and EC

Miao et al. identified shared DEGs between PCOS and EC. The comparison of the GSE48301 dataset, exploring DEGs between endometrial cell populations obtained from PCOS women (*n* = 6) and healthy controls (*n* = 6), with the GSE115810 dataset, identifying DEGs between normal human endometrium (*n* = 3) and malignant human endometrium derived from EC patients (*n* = 24), revealed 192 DEGs in common between the datasets. Amongst these DEGs, the analysis identified 10 top hub genes, highly connected genes to other genes in a network, enriched in the ribosome pathway (Table 2), which is consistent with the idea that overactive ribosome production is linked to the development of tumours. Experimentally verified interactions in the NetworkAnalyst platform presented four transcription factors indicating a strong correlation with the hub genes, namely, *KLF9* (Krüppel‐like factor 9), *PHF8* (PHD finger protein 8), *KDM5B* (lysine demethylase 5B) and *SAP30* (Sin3‐associated protein 30), all of which have been identified to have either a positive or negative role in cancer growth [9].

Shafiee et al. investigated the impact of insulin signalling in the development of EC in individuals with PCOS by comparing the expression levels of three vital genes involved in the insulin signalling pathway, namely, *IGF1* (insulin‐like growth factor 1), *PTEN* (phosphatase and TENsin homologue) and *IGFBP1* (insulin‐like growth factor binding protein 1). Expression at mRNA levels of these genes was compared in endometrial tissue taken from three groups of women: individuals with PCOS, EC patients and control (non‐PCOS women without EC). Results showed increased expression levels of these genes in individuals with EC or PCOS compared to controls, independent of BMI, waist–hip ratio, oestradiol and androgen levels (*p* < 0.01). The study also found significantly higher concentrations of the proteins in the serum of individuals with PCOS or EC compared to controls (*p* < 0.01) (Table 2) [8].

SREBP1 (sterol regulatory element binding protein‐1) plays a vital role in fatty acid synthesis and metabolism, as well as activating the phosphatidylinositol‐4, 5‐bisphosphate 3‐kinase pathway, which promotes cell proliferation. Studies have revealed abnormal regulation of intracellular lipogenesis in EC due to upregulation of *SREBP1*. This pathway may play a role in modulating the risk of EC in women with PCOS. Therefore, a study was conducted at Nottingham University Hospital with 102 women, including 34 participants with PCOS, 34 EC patients and 34 controls. The results indicated that SREBP1 expression at mRNA levels in endometrium tissue (*p* < 0.0001) and serum protein concentration (*p* < 0.05) was significantly higher in PCOS and EC patients than in controls (Table 2) [13].

A study at Nottingham University Hospital used RNA sequencing and identified 94 shared DEGs in endometrial biopsies, age‐ and BMI‐matched, from individuals with PCOS and EC patients relative to controls, with 12 genes being upregulated and 82 genes downregulated (*p* < 0.05) [28]. Significantly enriched gene networks were found to be involved in microtubule motor activity and cilia function. For more validation, expression levels of 94 DEGs were examined in EC patients using the TCGA (The Cancer Genome Atlas) database. Fourteen genes, including NQO1 (NAD(P)H quinone oxidoreductase 1), showed altered expression in EC compared to noncancerous endometrial specimens. NQO1 activates certain pharmaceuticals, the compounds manufactured for use as medicinal drugs, in the endometrium and protects proteins involved in proliferation, like p53, from degradation. Its upregulation has been reported in many human solid tumours, and high levels are associated with poorer patient prognosis. This suggests that tumours have the potential to bypass the antiproliferative effect of NQO1. Further investigation was conducted on NQO1 in the TCGA‐UCEC (uterine corpus endometrial carcinoma) dataset, indicating significantly higher expression of NQO1 and its target, p53 mRNA, in primary endometrial cancer specimens (*n* = 370) than in nontumour specimens (*n* = 11). The levels of both NQO1 and *TP53* gene expression were measured in a validated cohort, which confirmed their increased expression in endometrial biopsies from women with PCOS (*N* = 25) or EC patients (*N* = 25) compared to the control group (*N* = 25) (*p* < 0.05). Additionally, IHC staining confirmed significantly enhanced protein expression of NQO1 in EC patients (*N* = 91) compared to the nonmalignant endometrial tissue (*N* = 6) in the Manchester cohort. The regulation of NQO1 expression by the oestrogen receptor‐α, oestrogen receptor‐β and progesterone, along with the contribution of aberrant oestrogen and progesterone signalling to EC risk, suggest the possible involvement of NQO1 in the oestrogen‐related links between EC and PCOS (Table 2) [28].

Desai et al. used the Gene Expression Omnibus (GEO) database to identify DEGs in endometrial cells of obese individuals with PCOS compared to obese control women. The study found 28 deregulated genes, including human *HHIP* (hedgehog‐interacting protein), *FGF10* (fibroblast growth factor 10) and *HGF* (hepatocyte growth factor), which are involved in cancer pathways and closely linked to EC and OVCA (Table 2) [29].

Women with PCOS may have a different metabolic and hormonal environment, leading to an endometrial ‘disease phenotype’ that affects gene expression of endometrial cells. A study compared gene expression in proliferative‐phase endometrium between obese/overweight individuals with and without PCOS. The study used microarray analysis to collect data and compare different types of endometrial cells, and the results were validated using qRT PCR and IHC. The study found that DEGs were independent of BMI and could potentially cause inflammatory effects. For example, endometrial stromal fibroblasts obtained from obese/overweight people with PCOS had upregulated inflammatory genes compared to obese/overweight controls, including *CCL2* (C‐C motif chemokine ligand 2) and *TNAIFP3* (TNF, and α‐induced protein 3). The study further explored the most upregulated genes, such as *IL‐8* (interleukin‐8), *SPRR3* (small proline‐rich protein 3) and *LCN2* (lipocalin 2) in endometrial mesenchymal stem cells between two groups. Overall, the study found that individuals with PCOS have markedly altered expression of proinflammatory cytokine and immune response‐related genes in different endometrial cell populations, indicating a possible endometrial disease phenotype (Table 2) [41].

Serum assessment is widely used to evaluate human health. Some of the abovementioned studies have shown a correlation between endometrium mRNA levels and serum levels of certain proteins independent of contributing factors such as BMI, oestradiol and androgen levels. Serum protein levels of IGF1, IGFBP1, PTEN and SREBP1 might be potential biomarkers to predict EC risk in individuals with PCOS. Further research is necessary to verify these findings.

#### 3.3.2 PCOS and OVCA

Kori et al. applied a comparative and integrative analysis of transcriptome datasets (GSE7463, GSE14407, GSE7305, GSE1615a, GSE1615b and GSE10946 samples from PCOS cases in women with low BMI) to determine molecular signatures of ovarian diseases, including PCOS, ovarian endometriosis and OVCA. Ovarian tissue was used for analysis in all datasets. Results displayed six shared upregulated genes and 31 common downregulated genes amongst the diseases (Table 2 & Table S3). A significant relationship was found between certain gene signatures including *STK17B* (serine/threonine kinase 17B), *ICAM1* (intercellular adhesion molecule‐1), *XIAP* (X‐linked inhibitor of apoptosis) and cancer (Table 2 & Table S3). Common pathways were also identified, including focal adhesion, adherens junction, MAPK signalling pathway and pathways in cancer. Additionally, the analysis found 14 shared, altered transcription factors in both PCOS and OVCA, including *TP53*, that are generally linked to cell cycle processes and cancer (Table 2) [7].

Salusin‐β is a bioactive peptide found in human plasma. It is linked to the size and higher levels of endometriomas in individuals with PCOS and endometriosis. Endometriomas are cystic lesions filled with endometrial fluid, mainly found in the ovaries. Zhang et al. further investigated the role of salusin‐β in OVCA using 57 paired ovarian cancer specimens and matched adjacent normal tissues. The qRT‐PCR results indicated that salusin‐β expression was significantly higher in OVCA specimens than in normal adjacent tissues and positively correlated with the FIGO stage (*p* = 0.007) and lymph node metastasis (*p* = 0.002). The levels of salusin‐β showed a negative correlation with overall survival rate (*p* < 0.001). Salusin‐β mRNA levels were higher in OVCA cell lines (SKOV3, IGROV1, A2780 and OVCAR3) than in normal human ovarian surface epithelial HOSE 6.3 cells. Salusin‐β promotes cell proliferation and epithelial‐to‐mesenchymal transition (EMT), accelerating cancer cell migration, invasion and metastasis. Salusin‐β is overexpressed in OVCA tissues and cell lines, and increased levels were found in individuals with PCOS. This suggests that salusin‐β may play a role in the link between PCOS and OVCA (Table 2) [30].

Zou et al. analysed the TCGA and GEO databases (GSE140082 and GSE34526) to identify potential key genes associated with PCOS and OVCA. They found 128 shared DEGs enriched in genes for cell adhesion proteins, indicating a possible association between PCOS and OVCA. Additionally, 12 out of 128 genes were linked to the prognosis of OVCA based on a univariate Cox regression model, prognostic correlation model, risk score formula and Kaplan–Meier analysis. The study used data from the TCGA database to find the correlation between 12‐gene signatures and OVCA patient prognosis. Results showed that *OGN* (osteoglycin) had a negative association with OVCA patient prognosis, while *JCHAIN* (joining chain of multimeric IgA and IgM), *GALNT6* (polypeptide N‐acetylgalactosaminyltransferase 6), *CXCL11* (C‐X‐C motif chemokine ligand 11) and *STAT1* (signal transducer and activator of transcription 1) positively correlated with patient progression. The study found that OGN was also closely linked to chemotherapy resistance and was involved in ferroptosis and m6A methylation modifications, which are involved in OVCA development and progression. The study suggests that OGN might represent a key biomarker for indicating a higher OVCA risk in individuals with PCOS (Table 2) [31].

Studies suggest measuring salusin‐β serum levels and or OGN expression levels in granulosa cells in individuals with PCOS could be a useful diagnostic biomarker for early detection of OVCA, but further research is needed to validate this finding.

### 3.4 Protein‐ and Proteomics‐Based Analysis

Recent studies have revealed the benefits of analysing protein–protein interactions (PPI) networks to identify molecular mechanisms involved in disease association (Table 3).

#### 3.4.1 PCOS and EC

Tian et al. conducted a case–control study to compare visfatin levels in the serum and endometrium of individuals with PCOS and healthy controls (Table 3). Results displayed a significant increase in visfatin expression in the endometrium of subjects with PCOS compared to the controls, which was positively correlated with higher levels of visfatin in the serum (*p* < 0.05). The study also found higher expression of p‐Akt (phosphorylated serine–threonine protein kinase 1) and p‐ERK1/2 (phosphorylated extracellular signal‐regulated kinase 1/2) in the endometrium tissues of women with PCOS, which was positively correlated with high visfatin expression in PCOS endometrium tissues (p‐Act, *r* = 0.225 and *p* = 0.015; p‐ERK1/2, *r* = 0.260 and *p* = 0.013). The study then compared visfatin levels in individuals with PCOS and endometrial lesions to those with PCOS but no endometrial lesions. The results showed that visfatin levels in serum (*p =* 0.010) and endometrium (*p* = 0.027) were higher in individuals with PCOS and endometrial lesions. Furthermore, individuals with PCOS and endometrial lesions had higher expression of p‐Akt (*p =* 0.018) and p‐ERK1/2 (*p* = 0.035) than those with normal endometrium. Visfatin induces human VEGF (vascular endothelial growth factor) and MMP‐2/9 (matrix metalloproteinase‐2/9) production via MAPK and PI3K/Akt signalling pathways. Thus, visfatin could potentially be a biomarker for the malignant transformation of endometrium in individuals with PCOS [11].

Paulson et al. evaluated the correlation between prolactin receptor expression and endometrial proliferation by assessing endometrial cell Ki67 protein levels in individuals with PCOS and controls. The findings indicated that those with PCOS had higher Ki67 expression levels in their endometrium relative to the BMI‐matched controls. Moreover, there was a direct association between endometrial immunostaining levels of prolactin receptor and Ki67. The study suggested that higher levels of prolactin receptor expression could contribute to increased proliferation and a higher risk of endometrial cancer in individuals with PCOS (Table 3) [32].

Giordano et al. investigated the effect of hormones and metabolic factors on endometrial homeostasis in women with PCOS using apoptotic and proliferative markers. Immunohistochemistry staining of the endometrial samples showed that individuals with PCOS had an imbalance in endometrial homeostasis: a lower percentage of cells staining positively for CASP3 (caspase 3) and FAS (*p* < 0.001), but a higher ratio of BCL2/BAX and Ki67/CASP3. Additionally, fasting insulin and DHEAS (dehydroepiandrosterone sulphate) levels were positively and negatively associated with the BCL2 (r = 0.571, *p* = 0.020) and CASP3 (r = −0.715, *p* = 0.002) proteins, respectively, in subjects with PCOS. The imbalance may contribute to the observed excessive endometrial proliferation without apoptosis compensation, which could increase the risk of endometrial cancer. But such a progression would require the additional drive of specific somatic mutations of oncogenes and tumour suppressor genes (Table 3) [33].

A study by Hu et al. found that women with PCOS had higher levels of androgen receptor protein, TLR2 (Toll‐like receptor 2), IRF‐7 (interferon regulatory factor‐7) and NFκB (nuclear factor κB) signalling, cytokine production and endometrial inflammation relative to controls. The study concluded that the increase in TLR4/IRF‐7/NFκB signalling was due to androgen, which caused cytokine synthesis and further increased endometrial inflammation in women with PCOS (Table 3) [34].

Given the strong link between serum evaluation and health, and the consistent relationship between visfatin serum levels and endometrial expression, further research is needed to assess visfatin serum protein as a predictor of endometrial cancer risk in women with PCOS. A systematic study of serum proteins by mass spectrometry could also identify additional potential serum biomarkers.

#### 3.4.2 PCOS and OVCA

Kori et al. identified highly connected proteins (hub proteins) in individuals with PCOS, ovarian endometriosis and OVCA by constructing PPI networks using DEGs and topological analyses. The top deregulated hub proteins in PCOS were MYC (Myc proto‐oncogene protein), MAPK1 (mitogen‐activated protein kinase 1), SUMO2 (small ubiquitin‐like modifier 2), SRC (SRC proto‐oncogene) and IKBKE (inhibitor of nuclear factor kappa B kinase subunit epsilon). Most identified proteins are known to be involved in cancer. MYC, MAPK1 and IKBKE are considered as proto‐oncogenes. ESR1 and SUMO1 (small ubiquitin‐like modifier 1) were identified as shared deregulated hub proteins in PCOS and OVCA (Table 3) [7]. ESR1 and ESR2 have been extensively investigated in breast cancer, where antioestrogen treatment is well established, but their therapeutic importance in OVCA treatment is less evident (Table 3) [35].

Ramly et al. conducted a study using PCOSBase, a curated medical database, and the MCODE algorithm to identify proteins related to PCOS and their associated diseases. The results displayed five PCOS‐related proteins which were also found in OVCA, namely, CDKN1B (cyclin‐dependent kinase inhibitor 1B), PPP1CC (serine/threonine‐protein phosphatase PP1‐gamma catalytic subunit), URI1 (unconventional prefoldin RPB5 interactor 1), SKP2 (S‐phase kinase‐associated protein 2) and BRCA1. The BRCA1 protein is commonly mutated in breast cancer and OVCA (Table 3) [36]. While there is no evidence of increased risk of PCOS in Li Fraumeni family patients with germline *TP53* mutations or *BRCA1/2* germline mutations, the findings raise questions about any connection between abnormal variants of these genes and PCOS.

Li et al. used proteomic profiling to compare the ovarian proteins of women with PCOS to those of the control group, patients with one normal ovary whose one ovary was resected to treat a unilateral benign ovarian teratoma or an ovarian cyst. The study identified 18 differentially expressed proteins, 13 upregulated and 5 downregulated in PCOS ovaries (Table 3). Both WB and IHC confirmed dysregulation of several selected proteins comprising upregulation of RBP1, HSP90B1, CALM1 (*p* < 0.01) and downregulation of ANXA6 (*p* < 0.05). Abnormal expression of HSP90B1 and CALM1 affects cellular processes such as proliferation, survival and apoptosis and is linked to PCOS (Table 3) [37].

Proteomic studies of PCOS are limited. Further investigation is required to assess the functional significance of reported protein changes in predicting the risk of OVCA and EC.

### 3.5 Metabolomics‐Based Analysis

Metabolomics is an active area of oncology research being studied for its potential to improve cancer detection, monitor response to treatment and evaluate prognosis. Altered metabolite profiles are associated with tumour growth and biochemical activity. Therefore, metabolomic analyses may offer a screening method for early diagnosis of precancerous cells in the endometrium, which could lead to early therapeutic intervention to prevent EC development or progression in PCOS patients (Table 3) [10].

#### 3.5.1 PCOS and EC

Glycoproteins and sulphated glycosaminoglycans play important roles in various tissues, including endometrium. Giordano et al. compared chondroitin sulphate and heparan sulphate in the proliferative endometrium of individuals with PCOS and controls. They found that heparan sulphate levels were significantly higher in the endometrium of individuals with PCOS than in controls (*p* = 0.03). Heparan sulphate enhances the binding of growth factors to surface receptors, and its elevated levels may predict future neoplasia risk (Table 3) [1].

Shafiee et al. studied the relationship between lipid metabolism imbalances and EC. The study included 102 women divided into three groups—PCOS, EC and control. The researchers analysed the lipids of endometrial tissues and plasma samples using liquid chromatography and high‐resolution mass spectrometry. They found a significant decrease in monoacylglycerol 24:0 and capric acid in the endometrial tissue samples from patients with PCOS and EC compared to controls. However, no mutual metabolite in plasma samples from PCOS and EC was found. Further research is required to evaluate the potential role of monoacylglycerol 24:0 and capric acid in the relationship between PCOS and EC (Table 3) [10].

Hyaluronic acid is important in cell differentiation, secretion of TNF‐alpha and inhibiting vascularisation and angiogenesis in endometrial lesions. A study by Santos et al. found that eumenorrheic women have higher levels of endometrial hyaluronic acid during the proliferative phase than those with PCOS (*p* < 0.01). Hyaluronic acid protects the endometrium against leucocyte invasion, growth factors and other mitogenic substances. Women with lower levels of hyaluronic acid may have less protection against tumour development (Table 3) [38].

Overall, further research is needed to clarify the potential role of metabolites in the possible link between PCOS and EC.

#### 3.5.2 PCOS and OVCA

Kori et al. compared transcriptome datasets and reporter metabolite analysis of DEGs in PCOS, OVCA and ovarian endometriosis. No common reporter metabolite was found. However, individuals with PCOS exhibited downregulated alanine, pyruvate and gangliosides (GD1a, GM1b), which can signal cancer initiation. Elevated ubiquinol, a potential biomarker for oxidative stress involved in inhibiting apoptotic pathways, was also observed in individuals with PCOS (Table 3) [7].

### 3.6 Epigenetics‐Based Biomarkers

Epigenetic changes regulate gene expression and contribute to the development of many diseases. They have been investigated as potential markers for diagnosis, prognosis and risk indicators. Thus, identifying these changes is crucial for managing diseases, especially cancer (Table 4).

#### 3.6.1 PCOS andEC

Exosomes found in the serum or urine of cancer patients can potentially be used as biomarkers for cancer diagnosis and prognosis. Che et al. identified differentially expressed miRNAs between women with PCOS and EC patients. The results revealed miR‐27a‐5p as the most significantly augmented miRNA in PCOS subjects' serum exosomes. In vitro transfection experiments on the Ishikawa and HEC‐1A EC cell lines indicated that miR‐27a‐5p could promote migration and invasion of the EC cells by downregulating SMAD4 (SMAD family member 4) via direct targeting. Therefore, miR‐27a‐5p levels in exosomes could be a potential blood‐based biomarker for predicting EC in individuals with PCOS (Table 4) [12].

Makrinou et al. studied DNA methylation in granulosa lutein cells of subjects with PCOS and age‐/BMI‐matched controls. Since smoking is known to have effects on global DNA methylation, all participants in the study were nonsmokers. They found 106 differentially methylated CpG sites (*p*‐values < 5.8 × 10^−8^), 48% hypomethylated and 52% hypermethylated, associated with 88 genes. These genes were identified to play critical roles in gene regulation, endocrine and metabolic functions, immune response, cell signalling, cell death and survival. The study validated the results using pyrosequencing targeted analysis of six specific CpG sites, with additional samples from individuals with PCOS and controls. The core pathway analysis showed ‘molecular mechanism to cancer’ as the most statistically significant pathway with pathologies similar to EC (Table 4) [39].

#### 3.6.2 PCOS and OVCA

Noncoding RNAs regulate gene expression and can be found in bodily fluids. Research has revealed that miRNAs are differentially expressed in ovarian cells, follicular fluid and the circulation of subjects with PCOS. McAllister et al. employed small RNA deep sequencing (next‐generation miR‐seq) combined with functional analyses to identify miRNAs responsible for increased androgen biosynthesis in theca cells of women with PCOS, compared to age‐matched normal cycling controls. Amongst 18 differentially expressed miRNAs (*p* < 0.05), miR‐130b‐3p was significantly downregulated (*p* < 0.001). miR‐130b‐3p can potentially target a truncated isoform of *DENND1A* (domain containing 1A), a functional PCOS GWAS candidate shown to mediate hyperandrogenism in PCOS theca cells. Additionally, miR‐130b‐3p is differentially expressed in malignant cells compared to normal cells (Table 4) [40].

miR‐155 promotes the growth and spread of cancer cells and is found in high levels in the ovaries and serum of individuals with PCOS. Xia et al. compared 20 individuals with PCOS to a control group of 20 women and found that miR‐155 expression was significantly higher in the PCOS group (*p* < 0.001). Transfection of KGN cells (a type of human cell derived from an ovarian granulosa cell tumour) with miR‐155 resulted in increased cell proliferation, migration and invasion (*p* < 0.01). They also showed that miR‐155 targets PDCD4 (programmed cell death 4), a tumour suppressor gene, which may contribute to the link between PCOS and OVCA (Table 4) [16].

The overexpression of LncRNA TMPO‐AS1 has been found to promote cancer progression in ovarian and cervical cancers. Hou et al. evaluated the role of TMPO‐AS1 in PCOS and its potential role in the association of PCOS with OVCA. In PCOS follicular fluid, upregulation of TMPO‐AS1 and premature miR‐355‐5p was observed, while mature miR‐355‐5p was downregulated (*p* < 0.05). The study found that transfection of the KGN cells and primary granulosa cells with TMPO‐AS1 led to a decrease in mature miR‐355‐5p, significantly increasing cell proliferation. Given that miR‐355‐5p functions as a tumour suppressor in OVCA, it might have a role in the association between PCOS and OVCA (Table 4) [18].

Circular RNAs (circRNA) are stable noncoding RNAs that may play a role in disease development, including PCOS and cancer. Xu et al. evaluated expression levels of circ_FURIN and its alleged target, miR‐423‐5p, in the ovarian cortex tissues of subjects with PCOS compared to controls. The study showed a significant increase in circ_FURIN and a decrease in miR‐423‐5p, which inhibits OVCA. The interplay between circ_FURIN and miR‐423‐5p was confirmed in a PCOS cell model, decreasing circ_FURIN suppressed proliferation and induced apoptosis. This suggests a possible connection between PCOS and OVCA through circ_FURIN and miR‐423‐5p (Table 4) [17].

### 3.7 Candidate Drugs Identified Based on the Shared Signatures

Abnormal gene expression in PCOS, EC and OVCA might contribute to the increased risk of EC or OVCA in PCOS. Deregulated genes may drive the transformation process and represent therapeutic targets to reduce the risk of EC and OVCA in individuals with PCOS.

Metabolic syndrome, commonly found in women with PCOS and characterised by obesity, hyperinsulinaemia and diabetes, may play a significant role in the development of EC. Improving insulin resistance with metformin can reduce the risk of EC. Metformin, an insulin‐sensitising agent, is effective in reversing endometrial hyperplasia. It can potentially prevent cell proliferation via activating AMPK (AMP‐activated protein kinase) in EC cells, weakening mTOR (mammalian target of rapamycin) overactivation resulting from IGF1 upregulation. Notably, overexpression of IGF1 in the endometrium of both PCOS and EC patients has been reported. Combined metformin treatment with MPA (medroxyprogesterone17‐acetate) has shown synergistically antiproliferative effects on EC cell lines [42]. Metformin reduces inflammation by targeting the IRF‐7/NFκB pathway in endometrial samples of individuals with PCOS [34]. A pre‐ and 3‐month postmetformin therapy study showed that metformin treatment increases transcriptional upregulation of the *TP53* tumour suppressor gene in individuals with PCOS (*p* = 0.016), which may reduce the risk of EC [43]. It is important to note that while metformin has been used to treat PCOS, it is not licensed for this indication.

Increased SREBP1 and NQO1 expression in the endometrium of PCOS and EC patients present potential therapeutic targets. SREBP1 overexpression leads to elevated endometrial lipogenesis and a higher risk of EC in PCOS patients. Lipid‐lowering drugs, used as monotherapy or combined with agents that target insulin resistance, may help treat PCOS and prevent EC. Fenofibrate, a PPARα (peroxisome proliferator‐activated receptor α) agonist used in clinical practice as a lipid‐lowering agent, was predicted as a candidate drug [13, 44].

Miao et al. found shared DEGs between PCOS and EC (Table 2) and predicted drugs to reduce EC risk. Fenofibrate was shortlisted among 10 candidate drugs and was chosen for further analysis due to its high combined score. Fenofibrate increases HDL (high‐density lipoprotein) levels and decreases LDL (low‐density lipoprotein), cholesterol and triglycerides levels. Using molecular docking analysis, it had a good binding interaction with three hub protein targets, JUN, ESR1 and UBE2I. Hub proteins are highly connected proteins in the interaction network. Deregulation of ESR1 was explored as a shared DEG in both PCOS and EC [7] or as a common differentially expressed protein in PCOS and OVCA [9]. Fenofibrate has been reported to have anticancer effects in various human cancers. It inhibits proliferation and enhances apoptosis in Ishikawa endometrial cancer cells. It also enhances the metabolism of fatty acids instead of glucose in the tumour microenvironment, reducing tumour progression [9]. Fatostatin is a promising drug for treating PCOS and preventing EC in women with PCOS by targeting SCAP (SREBP cleavage‐activating protein) and inhibiting fatty acid synthesis. It has also been found to inhibit prostate cancer growth [44]. Several approved investigational and experimental candidate drugs with anti‐inflammatory and anticancer effects and low side effects, such as melatonin, resveratrol and quercetin, have also been found to have activity against ESR1 [45].

### 3.8 Conclusion

This study found that individuals with PCOS have genetic, epigenetic and other changes in their endometrium and ovary, which are relevant to the development of EC and OVCA. The study suggests that these changes can be used as biomarkers to identify individuals at risk of EC and OVCA and guide preventive drug therapies. Using minimally invasive serum samples, certain protein levels, such as IGF1, IGFBP1, SREBP1 and visfatin, could be used as potential biomarkers for the increased risk of EC and salusin‐β for malignant transformation of ovaries in individuals with PCOS. *TP53, BRCA1* and *ESR1* genes are frequently deregulated in the link between PCOS and OVCA. However, further studies are required to investigate how these common molecular signatures might translate to measurable plasma changes. EC and OVCA need to be added to the range of long‐term health consequences of PCOS and warrant increased surveillance among these patients. After a confirmatory diagnosis of PCOS, regular treatment and follow‐up are necessary to prevent or minimise the risk of future EC and OVCA or to detect these diseases as early as possible. Overall, further experiments and clinical trials are needed to confirm molecular signatures and potential drugs for preventing EC and OVCA in women with PCOS.

The study's strengths include a comprehensive literature search, a rigorous selection of research articles by two reviewers using a standard method and an assessment of publication bias. However, limitations include reference to studies with small sample sizes, different diagnostic criteria for PCOS detection or lack of reasonable adjustment for confounding covariables.

## Author Contributions

**M. Zanjirband:** conceptualization (lead), formal analysis (equal), investigation (equal), methodology (equal), project administration (lead), supervision (lead), validation (equal), visualization (lead), writing – original draft (lead), writing – review and editing (equal). **M. H. Nasr‐Esfahani:** conceptualization (supporting), writing – review and editing (equal). **N. J. Curtin:** writing – review and editing (equal). **Y. Drew:** writing – review and editing (equal). **S. Sharma Saha:** writing – review and editing (equal). **P. Adibi:** formal analysis (supporting), investigation (equal), methodology (equal). **J. Lunec:** conceptualization (supporting), validation (equal), writing – review and editing (equal).

## Conflicts of Interest

The authors declare no conflicts of interest.

## Supporting information


**Figure S1.** Risk of bias graph: Review of authors’ judgements about each of bias item presented as percentages across all included studies.

**Table S1.** Databases were searched using specific terms to find the articles regarding the correlation of PCOS with EC or OVCA.

**Table S2.** The citation, country, study design, sample size, and outcomes evaluated for each included study.

**Table S3.** A list of differentially expressed genes (DEGs) based on the transcriptomics analysis. Down‐regulated genes are shown as bold.

## Data Availability

Data available on request from the authors.
