# Supplementary figures and images for: A Systematic Review of the Molecular Mechanisms Involved in the Association Between PCOS and Endometrial and Ovarian Cancers
Source: J Cell Mol Med. 2024 Dec 25;28(24):e70312. doi: 10.1111/jcmm.70312 (PMC11669186; doi:10.1111/jcmm.70312)

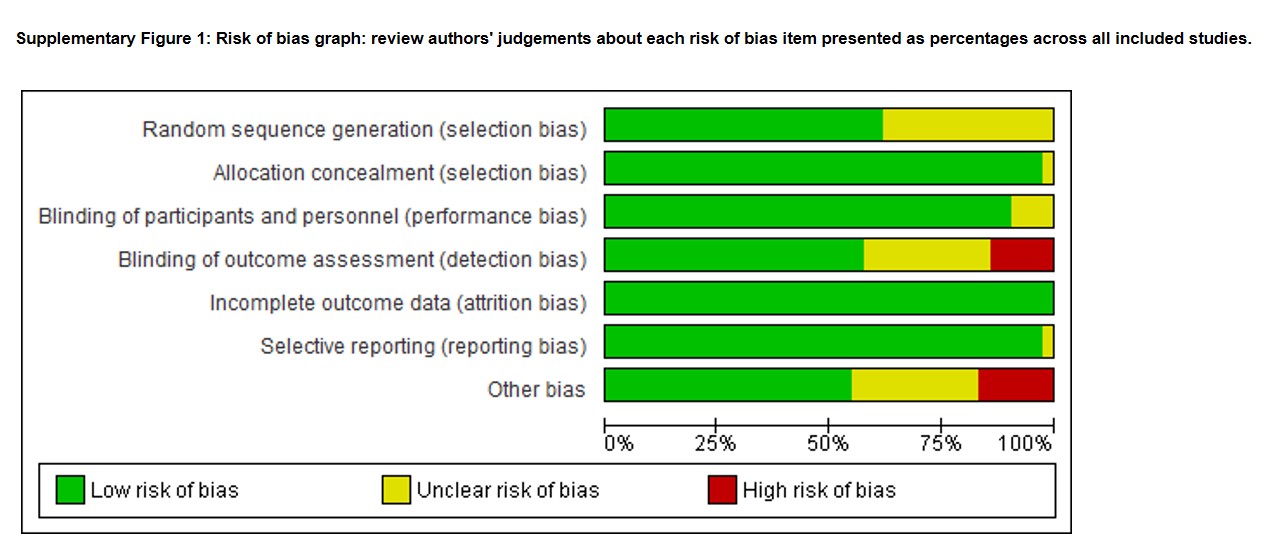

Supplement: Supplementary file 1 — Figure S1. Risk of bias graph: Review of authors’ judgements about each of bias item presented as percentages across all included studies. [file JCMM-28-e70312-s002.jpg]
