# Supplementary material for: A Systematic Review of the Molecular Mechanisms Involved in the Association Between PCOS and Endometrial and Ovarian Cancers
Source: J Cell Mol Med. 2024 Dec 25;28(24):e70312. doi: 10.1111/jcmm.70312 (PMC11669186; doi:10.1111/jcmm.70312)
Supplement: Supplementary file 2 — Table S1. Databases were searched using specific terms to find the articles regarding the correlation of PCOS with EC or OVCA. [file JCMM-28-e70312-s004.docx]

**Supplementary Table 1:** Databases were searched using specific terms to find the articles regarding the correlation of PCOS with EC or OVCA.

| No. | Databases/  Manual search | Search Terms  (Key Words) | Results | Excluded  articles | Included  Articles * |
| --- | --- | --- | --- | --- | --- |
| 1 | PubMed | “Gene signature and PCOS/polycystic ovarian syndrome and endometrial cancer/ovarian cancer”, “Transcriptome and PCOS/polycystic ovarian syndrome and endometrial cancer/ovarian cancer”, “Biomarker and PCOS/polycystic ovarian syndrome and endometrial cancer/ovarian cancer”, “Epigenetics and PCOS/polycystic ovarian syndrome and endometrial cancer/ovarian cancer”, “miRNA/micro-RNA and PCOS/polycystic ovarian syndrome and endometrial cancer/ovarian cancer”, “Methylation and PCOS/polycystic ovarian syndrome and endometrial cancer/ovarian cancer”, “Exosomes and PCOS/polycystic ovarian syndrome and endometrial cancer/ovarian cancer” | 408 | 377 | 31 |
| 2 | POPLINE | The same as above. | 454 | 427 | 27 |
| 3 | Manual Search | _ | 15 | 0 | 15 |
| 3 | Total | _ | 878 | 804 | 73 |
| 4 | Minus common in both | _ | _ | 13 | 60 |
| 7 | Minus ineligible study | _ | _ | 15 | 45 |

*: The excluded articles, including irrelevant articles, editorials, uninterpretable data, and reviews, were subtracted from the total number of articles found through searching on each dataset. Then, after the subtraction of excluded articles and the addition of manual search, the total number of 73 articles was considered eligible. Finally, after subtracting shared articles in both databases and further ineligible studies, 45 articles were included in this study.
