# Supplementary material for: A Systematic Review of the Molecular Mechanisms Involved in the Association Between PCOS and Endometrial and Ovarian Cancers
Source: J Cell Mol Med. 2024 Dec 25;28(24):e70312. doi: 10.1111/jcmm.70312 (PMC11669186; doi:10.1111/jcmm.70312)
Supplement: Supplementary file 3 — Table S2. The citation, country, study design, sample size, and outcomes evaluated for each included study. [file JCMM-28-e70312-s003.docx]

**Supplementary Table 2:** The citation, country, study design, sample size, and outcomes evaluated for each included study.

| **Author (Year) (Reference) ^a^** | **Country** | **Study design/Sample size** | **Cancer risk/Potential biomarker (s)/drug repositioning** |
| --- | --- | --- | --- |
| Giordano *et al.* (2015) (1) | Brazil | Case-control (Endometrial samples)  PCOS (10) VS control (8) | Endometrial heparin sulfate |
| Sekar *et al.* (2015) (2) | India | Case-control (Buccal, dermatoglyphics, blood samples)  PCOS (10), Age- & sex-matched control (4) | Chromosome aberrations |
| Ding *et al.* (2018) (3) | Taiwan | Observational Study  PCOS (8155), Control (32620) | Increased risk of EC in women with PCOS |
| Jia *et al.* (2020) (4) | China | Case report  EC (229), Patients also presented with PCOS (9) | 4% of EC patients presented PCOS  as well |
| Jiao *et al.* (2019) (5) | China | Case-control (ovarian tissue)  PCOS with irregular menstruation (10)  PCOS with regular menstruation (10) | Hypomethylation at oncologically significant regions, aberrant expression of cancer-related genes & miRNAs |
| Cirillo et al. (2016) (6) | USA | Prospective study (15,528) | A higher risk of OVCA for women with irregular cycles |
| Kori *et al.* 2016 (7) | Turkey | Case-control (bioinformatics analysis)  A total of 92 (46 diseased, 46 control ^b^) | DEGs, hub proteins, metabolic signatures & miRNAs signatures |
| Shafiee *et al.* (2016) (8) | UK | Cross-sectional (blood and endometrial samples)  EC (34), PCOS (34), Control (34) | DEGs & serum levels of their proteins |
| Miao *et al.* (2022) (9) | China | Case-control (bioinformatics analysis)  GSE48301: PCOS (6) VS control (6),  GSE115810: EC (24) VS control (3) | Common DEGs (192)  Common shared hub DEGs (10) |
| Shafiee *et al.* (2020) (10) | UK | Cross-sectional (blood and endometrial samples)  EC (34), PCOS (34), Control (34) | Monoacylglycerol 24:0 & capric acid |
| Tian *et al.* (2020) (11) | China | Case-control (blood specimens)  PCOS (80) VS control (80)  & (endometrial samples)  PCOS (60) VS control (60) | Protein levels of Visfatin, p-Akt & p-ERK1/2 |
| Che *et al.* (2020) (12) | China | Observational cohort (serum samples)  Exosomal miRNA sequencing: PCOS (4), Control 4)  Validated cohort: PCOS (30), Control (10) | Exosomal levels of miR-27a-5p |
| Shafiee *et al.* (2017) (13) | UK | Cross-sectional (serum and endometrial samples)  EC (34), PCOS (34), Control (34) | *SREBP1* expression  Serum protein levels of SREBP1 |
| Gottschau *et al.* (2015) (14) | Denmark | Observational cohort (endometrial tissue) PCOS (12,070) | A higher risk of EC for PCOS |
| Kokosar *et al.* (2016) (15) | Sweden | Case-control (sub-cutaneous adipose tissue biopsies)  Cohort 1: PCOS (64) Control (30)  Cohort 2: PCOS (21) Control (21) | Multiple transcriptional and epigenetic modifications are relevant to cancer development |
| Xia *et al.* (2020) (16) | China | Case-control (ovarian cortical tissues)  PCOS (20), control (20) | miR-155 |
| Xu *et al.* (2022) (17) | China | Case-control (ovarian cortex tissues)  PCOS (21), Control (13) | Circ_FURIN & miR-423-5p |
| Hou *et al.* (2021) (18) | China | Case-control (follicular fluid)  PCOS (60), Control (60) | Expression levels of LncRNA TMPO-AS1 |
| Fearnley *et al.* (2010) (19) | Australia | Case-control  EC (156, 12% of whom reported to have PCOS as well), Control (398) | A higher risk of EC for PCOS |
| Ye *et al.* (2019) (20) | Sweden | Cohort (females aged between 15 to 50 years)  (n = 3,493,604) | Excess cancer risk between PCOS and EC/OVCA |
| Park *et al.* (2011) (21) | Korea | Observational cohort (endometrial tissue) PCOS (117) | A higher risk of hyperplasia & EC |
| Wang *et al.* (2012) (24) | China | Case-control (blood samples)  EC (96), Control (192) | SNPs rs2479106 and rs13405728 |
| Day *et al.* (2015) (25) | UK | Case-control  PCOS (2,045), Control (98,886) | Six signals for PCOS at genome-wide statistical significance in/near 6 genes |
| Xiong *et al.* (2020) (26) | China | Meta-analysis of 45 case-control studies | MTHFR C677T & MTHFR A1298C polymorphisms |
| Bhanoori *et al.* (2018) (27) | India | Case-control  PCOS (110), Control (130) | *TP53* (rs1042522 G/C) & *BRCA1* (rs71361504 −/GTT, rs3092986 T/C)  polymorphisms |
| Atiomo et al. (2017) (28) | UK | Cross-sectional (endometrial biopsies)  qRT-PCR: PCOS (26), EC (25), control (25)  RNAseq: One woman from each arm | RNAseq: Common DEGs (94)  Validation using TCGA: 14 out of 94  qRT-PCR: DRGs (3) |
| Desai *et al.* (2017) (29) | India | Case-control (bioinformatics analysis)  (Endometrium mesenchymal/stromal  /endothelial/epithelial cells)  Obese PCOS (14), Obese control (15) | DEGs (5) |
| Piltonen *et al.* (2013) (30) | USA | Prospective Case-control (proliferative-phase endometrial tissue), Overweight/obese PCOS (6),  Overweight/obese Control (6) | DEGs (12) |
| Zhang *et al.* (2017) (31) | China | Case-control  OVCA (57), Matched adjacent normal tissues (57) | salusin-β |
| Zou *et al.* (2022) (32) | China | Case-control  TCGA (374 OVCA samples), GTEX database (88 normal ovarian samples), GSE34526 (PCOS granulosa cells 7 VS normal granulosa cells 3), GSE140082 (380 OVCA Samples) | DEGs (12) |
| Paulson *et al.* (2020) (33) | Sweden | Cross-sectional (blood and endometrial biopsies)  Obese-PCOS (2O), Obese-control (10)  NW-PCOS (11), NW-control (11) | Prolactin receptor |
| Giordano *et al.* (2022) (34) | Brazil | Cross-sectional (endometrial biopsies)  PCOS (11), Normal cycling non-PCOS controls (8) | DEPs (6) & homeostasis indexes |
| Hu *et al.* (2021) (35) | Sweden & China | Case-control (endometrial biopsies)  PCOS (22), non-PCOS (17) | DEPs (4) |
| Englert-Golon *et al.* (2021) (36) | Poland | Case-control (ovarian tissue)  OVCA (25), Control (27: 12 healthy control & 15 women with non-cancerous benign changes) | ESR1, ESR2, PELP1 & c-SRC |
| Ramly *et al.* (2019) (37) | Malaysia | Protein–Protein Interaction Network Analysis  8185-PCOS- related proteins | 5 Proteins involved in PCOS was found in OVCA |
| Zhang et al. (2016) (38) | China | Case-control (ovarian biopsies)  PCOS (10), control (10) | DEPs (18) |
| Simoes *et al.* (2019) (39) | Brazil | Case-control (endometrial biopsies)  PCOS (30), control (30) | Hyaluronic acid |
| Makrinou *et al.* (2020) (40) | UK | Case-control (follicular aspiration)  PCOS (16), Control (16)  Validated cohort: PCOS (42), Control (43) | Differentially methylated CpG sites (106) |
| McAllister *et al.* (2019) (41) | Columbus | Case-control (theca cells)  PCOS (7), Control (7) | Differentially expressed miRNAs relevant to OVCA signalling (18) |
| Xie *et al.* (2011) (42) | China | (EC cell lines) | Combination of metformin with MPA |
| Shafiee et al. (2015) (43) | Malaysia | Pre-post study without control  PCOS (40) | TP53 |
| Li *et al.* (2012) (44) | China & USA | Case-control (endometrial tissues)  EC (121), control (adjacent normal tissues were included for some cancer tissues.) | SREBP1 |
| Zanjirband *et al.* (2023) (45) | Iran | Case-control (bioinformatic analysis, granulosa cells)  PCOS (4), control (4) | Tamoxifen, melatonin, resveratrol, raloxifene, and quercetin |

a: References 22 & 23 are not included in Supplementary Table 1 because they relate to the methods, PRISMA and QUADAS-2.

b: GSE7463 & GSE14407: OVCA (21), non-OVCA (22); GSE7305: Ovarian endometriosis (10), Normal endometrium (10); GSE1615a, GSE1615b & GSE10946-lean samples: PCOS (15), Control (14).

DEGs, Differentially Expressed Genes; DEPs, Differentially Expressed Proteins; EC, Endometrial Cancer; GEO: Gene Expression Omnibus; MPA, MedroxyProgesterone17- Acetate; NW-PCOS, Normal Weight- PCOS; NW-control, Normal Weight-control; OVCA, Ovarian Cancer; p-Akt, Phosphorylated- Serine-Threonine Protein Kinase 1; p-ERK1/2, Phosphorylated- Extracellular Signal-Regulated Kinas1/2; PCOS, Polycystic Ovarian Syndrome; PRLR, Prolactin Receptor; SREBP1, Sterol regulatory element binding protein-1; TCGA, The Cancer Genome Atlas.
