# Supplementary material for: A Systematic Review of the Molecular Mechanisms Involved in the Association Between PCOS and Endometrial and Ovarian Cancers
Source: J Cell Mol Med. 2024 Dec 25;28(24):e70312. doi: 10.1111/jcmm.70312 (PMC11669186; doi:10.1111/jcmm.70312)
Supplement: Supplementary file 4 — Table S3. A list of differentially expressed genes (DEGs) based on the transcriptomics analysis. Down‐regulated genes are shown as bold. [file JCMM-28-e70312-s001.docx]

**Supplementary Table 3:** A list of differentially expressed genes (DEGs) based on the transcriptomics analysis. Down-regulated genes are shown as bold.

| **Author** | **Genes symbol** | **Genes name** |
| --- | --- | --- |
| Jiao *et al.* (5) |  |  |
|  | *ATP5F1* | F-type ATPases |
|  | *AURKA* | Aurora kinase A |
|  | *BMI1* | B-cell-specific Moloney murine leukaemia virus integration region 1 |
|  | *CASP2* | Caspase 2 |
|  | *CDC20* | Cell Division Cycle 20 |
|  | *DKC1* | Dyskerin pseudouridine synthase 1 |
|  | *ERCC3* | ERCC Excision Repair 3 |
|  | *GPD2* | Glycerol-3-Phosphate Dehydrogenase 2 |
|  | *MCM2* | Minichromosome Maintenance Complex Component 2 |
|  | *SKP2* | S-phase kinase-associated protein-2 |
|  | *STMN1* | Stathmin 1 |
|  | *TBX2* | T-Box Transcription Factor 2) |
| Kori *et al.* (6) |  |  |
|  | ***CBFB*** | **Core-binding factor subunit beta** |
|  | ***CCDC176*** | **Coiled-coil domain containing 176** |
|  | ***CENPV*** | **Centromere Protein V** |
|  | ***CHP1*** | **Calcineurin B homologous protein 1** |
|  | ***CLIC4*** | **Chloride intracellular channel 4;** |
|  | ***CMTM3*** | **CKLF-like MARVEL transmembrane domain-containing protein 3** |
|  | ***CRNDE*** | **Colorectal Neoplasia Differentially Expressed** |
|  | ***ELAVL1*** | **ELAV-like protein 1** |
|  | ***ESR1*** | **Estrogen Receptor 1** |
|  | ***FAM213A*** | **Family with Sequence Similarity 213, Member A** |
|  | ***FBXO28*** | **F-box only protein 28** |
|  | ***HERPUD1*** | **Homocysteine-responsive endoplasmic reticulum-resident ubiquitin-like domain member 1** |
|  | ***JAZF1*** | **Juxtaposed with another zinc finger protein 1** |
|  | ***MAG1*** | **Myelin-associated glycoprotein 1** |
|  | ***MAPKAP1*** | **MAPK Associated Protein 1** |
|  | ***NID1*** | **Nidogen-1** |
|  | ***NSF*** | **N-ethylmaleimide-sensitive factor** |
|  | ***PROSER1*** | **Prospero Homeobox Protein 1** |
|  | ***PSPC1*** | **Paraspeckle Component 1** |
|  | ***RAPGEF2*** | **Rap Guanine nucleotide Exchange Factor 2** |
|  | ***SERF2*** | **Small EDRK-Rich Factor 2** |
|  | ***SHROOM3*** | **Shroom Family Member 3** |
|  | ***SUZ12*** | **SUZ12 Polycomb Repressive Complex 2 Subunit** |
|  | ***TMOD3*** | **Tropomodulin 3** |
|  | ***TPD52L1*** | **Tumor Protein D52 Like 1** |
|  | ***TRIM33*** | **Tripartite Motif Containing 33** |
|  | ***UBE2J1*** | **Ubiquitin Conjugating Enzyme E2 J1** |
|  | ***XIAP*** | **X-linked Inhibitor of Apoptosis** |
|  | ***ZHHC2*** | **Zinc Finger CCHC-Type Containing 2** |
|  | ***ZFP36L2*** | **ZFP36 Ring Finger Protein Like 2** |
| Atiomo *et al.* (24) |  |  |
|  | *DNAJC15* | DnaJ Heat Shock Protein Family (Hsp40) Member C15 |
|  | *ESPN* | ESPIN |
|  | *FOXJ1* | Forkhead box protein J1 |
|  | *GJB2* | Gap Junction Beta 2 |
|  | *IFI6* | Interferon-alpha Inducible Protein 6 |
|  | *IFI27* | (Interferon α-Inducible Protein 27 |
|  | *KRT5* | Keratin 5 |
|  | *LGR5* | Leucine-rich repeat-containing G-protein coupled receptor 5 |
|  | *MUC16* | Mucin 16, Cell Surface Associated |
|  | *NQO1* | NAD(P)H Quinone Oxidoreductase 1 |
|  | *PLEKHS1* | Pleckstrin homology domain-containing S1 |
|  | *RSPH1* | Radial spoke head 1 homolog |
|  | *S100A8* | S100 Calcium Binding Protein A8 |
|  | *SLPI* | Secretory Leukocyte Protease Inhibitor |
